# The value of extracorporeal photopheresis as an immunosuppression-modifying approach in solid organ transplantation: a potential solution to an unmet medical need

**DOI:** 10.3389/fimmu.2024.1371554

**Published:** 2024-05-23

**Authors:** Jean-François Augusto, Christian Benden, Fritz Diekmann, Andreas Zuckermann

**Affiliations:** ^1^Department of Nephrology-Dialysis-Transplantation, University Hospital of Angers, Angers, France; ^2^Faculty of Medicine, University of Zurich, Zurich, Switzerland; ^3^Renal Transplantation Unit, Department of Nephrology and Kidney Transplantation, Hospital Clinic, Barcelona, Spain; ^4^Department of Cardiac Surgery, Medical University of Vienna, Vienna, Austria

**Keywords:** allograft rejection, calcineurin inhibitors, extracorporeal photopheresis, immunosuppression, solid organ transplantation

## Abstract

Allograft rejection is a critical issue following solid organ transplantation (SOT). Immunosuppressive therapies are crucial in reducing risk of rejection yet are accompanied by several significant side effects, including infection, malignancy, cardiovascular diseases, and nephrotoxicity. There is a current unmet medical need with a lack of effective minimization strategies for these side effects. Extracorporeal photopheresis (ECP) has shown potential as an immunosuppression (IS)-modifying technique in several SOT types, with improvements seen in acute and recurrent rejection, allograft survival, and associated side effects, and could fulfil this unmet need. Through a review of the available literature detailing key areas in which ECP may benefit patients, this review highlights the IS-modifying potential of ECP in the four most common SOT procedures (heart, lung, kidney, and liver transplantation) and highlights existing gaps in data. Current evidence supports the use of ECP for IS modification following SOT, however there is a need for further high-quality research, in particular randomized control trials, in this area.

## Highlights

- Allograft rejection is an ongoing issue following solid organ transplantation.
- Current standard of care IS therapies have burdensome side effects.
- Extracorporeal photopheresis has IS-modifying potential.
- Extracorporeal photopheresis may allow lower standard IS management strategies.

## Introduction

Solid organ transplantation (SOT) is currently considered a life-saving or life-prolonging intervention for patients with irreversible organ damage or end-stage organ failure, providing appropriate outcomes in selected patients (1, 2). As well as promoting survival, SOT also aims to improve patient health-related quality of life (HRQoL) (1, 3). In 2015, ~127,000 SOTs were reported worldwide, including ~41,000 in Europe. The most commonly performed SOT procedures worldwide were kidney (66.6%), liver (21.9%), heart (5.5%), and lung (4.0%) transplantation (4).

The risk of allograft rejection is a critical issue post-transplant that varies by SOT type. Rejection can be classified as acute or chronic, and either T-cell- or B-cell-mediated; acute-on-chronic and mixed T/B-cell rejection have also been reported (5–7). To reduce the risk of graft rejection, transplant recipients may receive induction immunosuppression (IS), such as anti-thymocyte globulins (ATG) or anti-IL-2 receptor antibodies (e.g., basiliximab) (7, 8). This is typically administered during the early post-operative period and followed by a long-term maintenance IS regimen (8–10). Similar maintenance immunosuppressive agents are delivered across all SOT types, with standard of care typically involving a calcineurin inhibitor (CNI), e.g., tacrolimus; an anti-metabolite, e.g., mycophenolate mofetil (MMF); and corticosteroids (CS), e.g., prednisone (8–10). In the event of allograft rejection, augmentation of the immunosuppressive regimen is generally needed, with high-dose or “pulse” CS therapy and short courses of ATG in selected cases of allograft rejection unresponsive to CS; this reflects mainly therapy against cellular rejection as compared to antibody-mediated rejection (11–15).

### Side effects of standard immunosuppression

While CNIs make up an essential component of current IS regimens, their use is frequently associated with acute and chronic renal impairment, post-transplant diabetes, hypertension, and dyslipidemia (16). The key strategy in minimizing this nephrotoxicity is to reduce CNI exposure to the minimal effective dosage. The use of mammalian target of rapamycin (mTOR) inhibitors, such as everolimus, or co-stimulation blockade with belatacept, can facilitate the reduction or withdrawal of CNI use, but is constrained by a delicate risk-benefit ratio and has not been examined in all organ types (17–20).

There is also a well-established link between IS and oncogenesis. Hanahan and Weinberg’s work classifying the hallmarks of cancer considers immune surveillance critical to identifying and destroying burgeoning tumors (21). Although this link may mean IS imposes a high risk of tumor development, the reality may not be so simple due to the complexity of the immune system and the possibility that it has dichotomous roles in both antagonizing and enhancing tumor development and progression (21).

Furthermore, IS treatments reduce the body’s defenses against opportunistic pathogens; this results in a burden of infective complications even in otherwise successful transplantations (20). Common infections include, but are not limited to, cytomegalovirus (CMV), *Pneumocystis jirovecii*, herpes simplex virus, BK virus, and *Mycobacterium tuberculosis* complex. With an increase in the number of infections produced by multi-drug resistant bacteria, these risks are only set to increase (20, 22).

While the use of standard IS minimizes the risk of acute or chronic graft failure and has been shown to reduce mortality among transplant patients, effective strategies to minimize these side effects remain a key unmet medical need for patients undergoing SOT (8, 23).

### Extracorporeal photopheresis

Extracorporeal photopheresis (ECP) is a leukapheresis-based therapy approved for the treatment of disorders with T-cell involvement, including cutaneous T-cell lymphoma, autoimmune diseases, and graft versus host disease (GvHD) following hematopoietic stem cell transplantation (HSCT) (24, 25). There is also evidence that ECP can be used as prophylaxis or treatment for solid organ allograft rejection (25–27), and it has shown potential as an IS-modifying therapy for patients undergoing SOT (24, 27).

The ECP procedure can be performed using a single integrated system (24). During treatment, the integrated system collects whole blood from the patient and separates leukocytes from plasma and non-nucleated cells. The leukocytes are then treated with a photosensitizing agent (8-methoxypsoralen) and exposed to ultraviolet-A (UVA) irradiation, before reinfusion (24, 25). After treatment, a large proportion of leukocytes undergo apoptosis, and subsequently phagocytosis by immature dendritic cells, promoting immunomodulatory effects (25, 28). The exact mechanisms of action by which ECP mediates its effects have not been fully characterized; however it is hypothesized that ECP works through a combination of dendritic cell initiation, modification of cytokine profiles and stimulation of T-cells (25). Generally, these activities start with the induction of apoptosis and simultaneous physiological activation of monocytes. These activated monocytes are presented to apoptotic lymphocytes and phagocytosed and, in the case of cancerous cells, leads to improved anti-tumor immunity (29–33). Additionally, and most relevant here, the immune tolerance aspects of the ECP mechanism of action are thought to be mainly actioned through Treg cell production and stimulation of anti-inflammatory cytokines (e.g. IL-10 and TGF-β) (25, 29, 34). These multiple effects of ECP, including anti-tumor immunity, inhibition of inflammation vis immune tolerance and modulation of genes involved in cell adhesion and diapedesis, work to restore healthy immune function by immune modulation (29–33); as such, it is important to note that, unlike conventional immunosuppressive therapies, ECP promotes regulatory T-cell production without inducing global IS (24, 26, 28).

The aim of this review is to discuss the IS-modifying effects of ECP in the field of SOT, with evidence from heart, lung, kidney, and liver transplantation, and the potential of ECP to augment conventional IS regiments in post-SOT patients.

## Summary of key evidence

A summary of the characteristics of the key studies included in this review can be found in **Table 1**.

**Table 1:** Summary of key studies.

Author and year	Study design	Level of Evidence^a^	Country	ECP treatment regimen	Patients (n)	Inclusion criteria	Outcome measures presented
Heart transplantation								
Barr et al. (1998) (35)	Multicenter, interventional, comparative, prospective RCT	3	United States and Europe	Month 1: 5 x 2 sessions on consecutive days Month 2–3: 2 x every other week Month 4–6: Q4W	60	Recipients of primary cardiac transplants	• Rejection episodes • Side effects • Mortality
Dall’Amico et al. (1995) (36)	Single-center, observational, single-arm, prospective case series	4	Italy	Month 1–6: 2 consecutive sessions Q4W	8	Patients experiencing RR	• Histological grading of rejection • Rejection episodes • Dose of standard immunosuppression • Side effects
Goekler et al. (2022) (37)	Single-center, interventional, single-arm, prospective case series	4	Austria	Month 1: 5 x 2 sessions on consecutive days Month 2–3: 2 x every other week Month 4–6: Q4W	28	Patients at high-risk of cancer recurrence or sepsis	• Histological grading of rejection • Side effects • Mortality
Maccherini et al. (2001) (13)	Single-center, observational, single-arm, prospective case series	4	Italy	Month 1: Twice per week Month 2–3: Q1W Month 4–5: Q1M Total ECP procedures: 20	12	Patients experiencing RR, recurrent infections associated with AR or moderate rejection	• Histological grading of rejection • Rejection episodes • Dose of standard IS • Side effects
Savignano et al. (2017) (38)	Single-center, observational, single-arm, retrospective case series	4	Italy	Median time from transplant to ECP: 18 months Median time range: 12 months Median ECP procedures: 24	8^b^	Patients experiencing RR/persistent rejection with coexistence of severe comorbidities	• Histological grading of rejection • Rejection episodes • Dose of standard IS • Side effects
Barten et al. (2023) (39)	Multicenter, observational, retrospective, single-arm chart review study	4	Austria, Germany, France, Hungary, and Italy	Mean treatment duration for patients with ongoing ECP treatment: 18.8 months Mean treatment duration for patients who completed ECP treatment: 9.0 months	102	Patients who started ECP treatment following heart transplantation	• Graft function • Response to ECP treatment • Complications • Overall survival • ECP-related safety
Lung transplantation								
Schoch et al. (1999) (40)	Single-center case study report	4	Switzerland	Month 1–4: twice per month (8 treatment cycles) Months 4–: Q4W	1	NA	• Reduction of cyclosporine dose • Histological grading of rejection • Treatment response measured by IL-10, surrogate for PTLD
Benden et al. (2008) (41)	Single-center, retrospective case series	4	Switzerland	ECP was performed on 2 consecutive days Q4W-Q6W for 12 cycles^c^	24	Lung transplant recipients experiencing BOS or acute RR	• Forced expiratory volume • Allograft survival • Side effects
Robinson et al. (2018) (42)	Case study	4	Switzerland	Patient underwent 10 ECP cycles within the first 74 days, ECP was then performed Q2W	1	NA	• Forced expiratory volume • Allograft function • Clinical status
Steinack et al. (2020) (43)	Single-center case study report	4	Switzerland	Month 1: Q1W (2 consecutive days) Month 2–5: Q2W Month 5–: Q4W	1	NA	• Dose of standard of care IS before and after ECP treatment • Allograft function and recovery
Leroux et al. (2022) (44)	Single-center study report	4	France	Month 1–6: Q2W (2 consecutive days) Month 6–: progressively extended to Q4W, Q6W and Q8W	12	Lung transplant recipients diagnosed with BOS	• Monthly FEV_1_ variation	
Kidney transplantation								
Dall’Amico et al. (1998) (45)	Single-center case series	4	Italy	Month 1: Q2W (consecutive days) Month 2–3: 2 treatments biweekly (consecutive days) Month 4–7: Q1M	4	Adolescent patients with RR episodes after renal transplantation resistant to standard IS	• Dose of standard of care IS before and after ECP treatment • Allograft function • Rejection episodes • Side effects
Kumlien et al. (2005) (46)	Single-center case series	4	Sweden	ECP Q2–3W Total range: 6–26 treatments	7	Patients with biopsy proven acute refractory rejections	• Allograft function • Serum creatine levels • Side effects
Jardine et al. (2009) (12)	Single-center, prospective case series	4	Australia	Month 1: ECP Q1W Month 2–5 (varied): ECP every other week Total range: 5–12 treatments	10	Renal transplant recipients with rejection resistant to standard IS	• Dose of standard of care IS before and after ECP treatment • Allograft function • Histological grading of rejection
Tamain et al. (2019) (47)	Multicenter retrospective case series	4	France	Month 1: 1–2 ECP sessions per week Month 2 onwards: ECP frequency gradually reduced to Q1M	33	Adult kidney transplant recipients with rejection, resistant to or contraindicated for standard therapies	• Allograft survival • Patient survival • Causes of graft loss
Augusto et al. (2021) (48)	Single-center case series	4	France	Standard ECP for 15–45 sessions	3	NA	• Kidney function • Donor specific antibody • Graft histology
Liver transplantation							
Urbani et al. (2007) (49)	Single-center, controlled trial (ECP group prospectively analyzed and controls retrospectively analyzed)	4	Italy	Month 1: Day 2, 6, then Q1W Then Q1W or Q1M dependent on transplant success	36 (18 per group)	Patients at risk of post-liver transplant renal impairment and neurological complications	• Treatment success rates (CNI-sparing or CNI-delayed regimen) • Time from LT to CNI introduction • Side effects

AR, acute rejection; BOS, bronchiolitis obliterans syndrome; CNI, calcineurin inhibitors; ECP, extracorporeal photopheresis; FEV_1_, forced expiratory volume

in one second; IL-10, interleukin-10; IS, immunosuppression; ISHLT, International Society for Heart and Lung Transplantation; LT, liver transplantation; NA, not available; PTLD, post transplantation lymphoproliferative disorder; Q1W, every week; Q2M, every two months; Q2W, every two weeks; Q4W, every four weeks; Q6W, every 6 weeks; RCT, randomized controlled trial; RR, recurrent rejection.

^a^Level of evidence based on the Oxford center for Evidence-Based Medicine 2011 Levels of Evidence in which Level 1 represents the highest quality evidence. Level 1: systematic review; Level 2: randomized/observational trial; Level 3: non-randomized controlled cohort; Level 4: case-series or historically controlled studies; Level 5: mechanism-based reasoning (50); ^b^One of the eight patients could not be evaluated; ^c^Two of the 24 patients were still completing the 12 cycles at end of study period.

### Heart transplantation

Cardiac allograft rejection is the major cause of morbidity and mortality in the first three years following heart transplantation (38), with acute rejection (AR) occurring in approximately 13% of patients within the first year (51). This explains the necessity of IS, however opportunistic infections, nephrotoxicity, neurotoxicity, and secondary malignancies have been linked to the use of standard IS in heart transplant recipients (38).

ECP has been used successfully to promote a more tolerogenic state in transplant patients and to modify the dosage of standard IS required by heart transplant recipients (27). The British Photodermatology Group (BPG), the UK Cutaneous Lymphoma Group (UKCLG; formerly the UK Skin Group) and published guidelines from the American Society for Apheresis (ASFA) support the use of ECP as a treatment for cardiac allograft rejection and rejection prophylaxis (26, 52, 53). The International Society for Heart and Lung Transplantation (ISHLT) also recommends ECP for the treatment of chronic or resistant acute cellular rejection (53–55).

#### ECP as rejection prophylaxis in heart transplantation

In 1998, Barr et al. published a randomized controlled trial (RCT) reporting on the incidence of AR and infection among heart transplant recipients who received ECP as prophylactic therapy starting the first month following transplant (35). A total of 60 patients were randomized to receive either standard triple immunosuppression (cyclosporine, azathioprine, and prednisone) or standard triple immunosuppression at the same dose plus ECP. After a 6-month follow-up period, a statistically significant reduction in the mean number of cardiac rejection episodes was observed in the ECP-treated arm (ECP treatment: 0.91 versus standard of care: 1.44, *P*=0.04). Significantly more ECP-treated patients (27 out of 33) experienced ≤1 rejection episode compared with the standard of care arm (14 out of 27; *P*=0.03). Of note, the study did not demonstrate a significant difference between the rates of infection in the two treatment arms. Therefore, these results indicated that adding ECP to standard IS led to improved outcomes in terms of rejection episodes and increased the degree of tolerance, while not leading to an increase in infections (38).

Since the publication of Barr et al, further evidence has been published supporting the use of ECP to reduce the risk of rejection with some demonstrating comparability to conventional corticoid therapy (35, 56). Kirklin et al. (2006) reported that in 36 patients who received at least three months of ECP following rejection with hemodynamic compromise, recurrent rejection or as prophylaxis in the presence of anti-donor antibodies, rejection risk was decreased (57).

More recently, Gökler et al. (2022) established a prophylactic ECP protocol for heart transplant recipients at high risk of cancer recurrence or post-operative infection. A total of 28 patients were treated with ECP immediately following heart transplantation in this single arm pilot cohort study (37). The main outcome of the study was one-year survival of patients, which was 88.5% (25/28) patients. All 28 patients avoided induction therapy, delayed the start of CNI treatment (by three to seven days post-transplant), delayed steroid therapy at a lower dose and lowered target levels of tacrolimus. The majority of patients did not experience rejection following heart transplant (median follow-up of 23.7 months [IQR 12.7–33.4]); only four out of 28 patients showed biopsy-proven signs of cellular rejection during or after ECP treatment and only one patient had biopsy-proven signs of antibody-mediated rejection. The infectious complication rate was reported to be ~18%. This may reflect the high-risk patient population of the study, as the biggest patient subgroup included patients with an established infection prior to transplantation. Overall, patients who received ECP avoided allograft rejection and reduced standard IS regimens.

#### ECP as rejection treatment for heart transplantation

The potential role of ECP has also been explored in heart transplant recipients experiencing allograft rejection. In two published prospective case series involving patients with acute recurrent rejection, ECP treatment allowed a reduction in the dose of conventional IS therapy, particularly CS (13, 36). Dall’Amico et al. (1995) found that prednisone, cyclosporine A, and azathioprine were reduced by 44%, 21%, and 29% respectively, following six cycles of ECP (36). Maccherini et al. (2001) reported that reduction of IS therapy was possible in all 12 patients following ECP treatment, though the dose reduction was not specified (13). Additionally, even though standard IS was reduced in both studies, patients treated with ECP experienced a substantial reduction in the number of rejection episodes, with no major side effects observed: Maccherini et al. (2001) found that ECP reduced the mean number of moderate to severe rejection episodes from 3 to 0.4 per patient, while Dall’Amico et al. (1995) reported that the number and severity of rejection episodes was reduced in seven out of eight treated patients (13, 36).

Recently, Barten et al. (2023) conducted a retrospective chart review study of 102 heart transplantation patients, which found that rejection ISHLT grades were reduced in 92.3% of patients who started ECP to treat acute cellular rejection, antibody-mediated rejection or mixed rejection (39). Additionally, in patients who initiated ECP to prevent rejection, 88.2% remained rejection-free, despite a reduction in IS therapy.

The potential of ECP as an IS-modifying treatment can also be seen in high-risk patients. In one retrospective study conducted by Savignano et al. (2017), patients with severe comorbidities were treated for recurrent or persistent rejection using ECP (38). The comorbidities included, but were not limited to, ulcerative colitis, malignancies, osteoporosis, diabetes mellitus, chronic kidney disease, CMV reactivation, and infections/infestations (cardiac toxoplasmosis, pulmonary aspergillosis, condylomatosis). The dose of standard IS was reduced in six out of eight patients studied (specific dose reductions not reported) following the addition of ECP, including in three patients who were classified as ECP non-responders based on biopsy results.

### Lung transplantation

A key threat to long-term survival and HRQoL following lung transplantation is chronic lung allograft dysfunction (CLAD). The most common CLAD phenotype is bronchiolitis obliterans syndrome (BOS) (27). Initial treatment of BOS may include augmentation of standard IS regimens, such as high-dose pulses of CS (while avoiding sustained administration of CS) and switching CNIs and drug classes (including the use of mTOR inhibitors) (58). Subsequent salvage therapy for those with unresponsive BOS includes ATG, and less frequently total lymphoid irradiation, OKT3, alemtuzumab, methotrexate, and cyclophosphamide (55). In selected cases, surgical treatment of gastroesophageal reflux disease may be considered. In most cases, this approach of modifying standard IS regimens seems to only stabilize or slow BOS progression, rather than show reversal of the process or normalization of graft function (27, 41). Thus, there is a need for more effective treatment options.

The ASFA published guidelines suggest ECP may be an appropriate treatment of lung transplant rejection, especially for those with early BOS (27, 59). The European Dermatology Forum indicated that ECP has been used in lung transplant recipients with a low rate of side effects, and that it can stabilize lung function in patients with CLAD/BOS (53).

Overall, ECP has shown promise in patients with acute recurrent cellular rejection and BOS, but there is a need for prospective RCTs in this specific field. While there are no guidelines or recommendations for early prophylactic use of ECP in lung transplantation, this indication is currently under investigation (53).

#### ECP as rejection treatment for lung transplantation

One of the earliest case reports on ECP used to modulate the dose of standard IS therapy for lung transplant rejection was presented by Schoch et al. in 1999 (**Table 2**) (40). In this case, a 17-year-old female patient with cystic fibrosis underwent bilateral lung transplantation, with subsequent diagnosis of post-transplantation lymphoproliferative disorder (PTLD). Cyclosporine was reduced from 250 to 100 μg/L, oral valaciclovir was increased to 4.5 g daily, and ECP was started twice monthly on a trial basis. The treatment was successful over the one-year observation period, with a positive response in the patient’s PTLD, following the immunomodulation and addition of ECP. Since publication of that case study, ECP has been used successfully for both acute cellular and chronic rejection (CLAD (41, 60), recurrent acute cellular rejection [ACR] (61) and primary graft dysfunction [PGD] (42)).

**Table 2:** IS--modulating outcomes from included studies.

Author and year	IS regimen at baseline	Change in standard IS regimen^a^
Heart transplantation		
Barr et al. (1998) (35)	Standard therapy alone (mean ± SD, n=27) • Cyclosporine, months 0–3: 4.6 ± 1.6 mg/kg/day • Cyclosporine, months 4–6: 4.7 ± 1.9 mg/kg/day • Azathioprine: dose NR • Prednisone: dose NR	Standard therapy plus ECP (mean ± SD, n=33) • Cyclosporine, months 0–3: 4.3 ± 1.1 mg/kg/day • Cyclosporine, months 4–6: 4.4 ± 1.2 mg/kg/day • Azathioprine: dose NR • Prednisone: dose NR
Dall’Amico et al. (1995) (36)	Maintenance therapy in 6 months preceding ECP therapy (mean ± SD, n=8) • Prednisone: 15.1 ± 4.7 mg/day • Cyclosporine: 361 ± 129 mg/day • Azathioprine: 134 ± 43 mg/day	Maintenance therapy during ECP (mean ± SD, n=8) • Prednisone: 9.4 ± 2.9 mg/day • Cyclosporine: 302 ± 129 mg/day • Azathioprine: 119 ± 55 mg/day
Goekler et al. (2022) (37)	Tacrolimus: dose NR Steroids: dose NR MMF: dose NR	No change reported
Maccherini et al. (2001) (13)	Standard triple immunosuppressive therapy (cyclosporine, azathioprine, steroids; n=6) Monotherapy with cyclosporine (n=2) Cyclosporine and steroids at 0.1 mg/kg (n=3)	Reduction of immunosuppression possible in all patients, especially steroids; specific doses NR
Savignano et al. (2017) (38)	Specific doses NR; standard triple immunosuppressive therapy consisted of the following: combination of prednisone/tacrolimus/MMF, prednisone/cyclosporin/everolimus or prednisone/cyclosporin/azathioprine	Reduction of standard immunosuppressive therapy (n/N=6/8)
Barten et al. (2023) (39)	Specific doses NR n (%) at the start of ECP treatment in patients with ongoing ECP treatment • Immunosuppressants: 47 (100%) • Rituximab: 2 (4.3%) • Steroids: 41 (87.2%) • Plasma exchange: 0 (0%) n (%) at the start of ECP treatment in patients with completed ECP treatment • Immunosuppressants: 58 (100%) • Rituximab: 1 (1.7%) • Steroids: 55 (94.8%) • Plasma exchange: 3 (5.2%)	Specific doses NR n (%) at the last reported visit in patients with ongoing ECP treatment • Immunosuppressants: 47 (100%) • Rituximab: 0 (0%) • Steroids: 37 (78.7%) • Plasma exchange: 0 (0%) n (%) at the last reported visit in patients with completed ECP treatment • Immunosuppressants: 58 (100%) • Rituximab: 0 (0%) • Steroids: 47 (81.0%) • Plasma exchange: 0 (0%)
Lung transplantation		
Schoch et al. (1999) (40)	Initial standard therapy • Cyclosporine: 250 μg/L • Azathioprine: dose NR • Prednisone: dose NR • Antithymocyte globulin: dose NR • Oral valaciclovir: 1.5 g/day A2 rejection treatment • Methylprednisolone: 1 g dose followed by 0.5 g/day for 2 days then tapered	• Cyclosporine: 100 μg/L • Oral valaciclovir: 4.5 g/day
Steinack et al. (2020) (43)	Induction therapy • Basiliximab Initial standard therapy • Cyclosporine: C2 target level 1200–1500 μg/L after 48 hours • MMF: 1.0 g bid • Methylprednisolone: 30 mg, as per center standard • Anti-infective treatment: dose NR	• Cyclosporine: C2 target level 180–200 μg/L • Methylprednisolone: 25 mg
Kidney transplantation		
Jardine et al. (2009) (12)	Pre-ECP rejection therapy (mean, n=10) • Steroid pulse: 4.775 g in total • Antilymphocyte: 15.4 days therapy • Standard maintenance immunosuppressive therapy	During ECP therapy • Steroid pulse: 1.05 g in total • Antilymphocyte: 1.7 days therapy Post-ECP therapy • Steroid pulse: 0.375 g in total • Antilymphocyte: 0 days therapy
Dall’Amico et al. (1998) (45)	Pre-ECP immune suppressive therapy (mean ± SD, n/N=3/4) • Prednisolone: 16 ± 11 mg • Cyclosporine A: 220 ± 58 mg/d • Azathioprine: 93 ± 12 mg/d	6 months post-ECP immune suppressive therapy (mean ± SD, n/N=3/4) • Prednisolone: 8.5 ± 1 mg • Cyclosporine A: 217 ± 40 mg/d • Azathioprine: 93 ± 12 mg/d
Liver transplantation		
Urbani et al. (2007) (49)	Initial standard therapy • Cyclosporine: 10 mg/kg/day • Tacrolimus: 0.1 mg/kg/day Antimetabolites and/or corticosteroids, as per center standard (dose NR)	• Spared CNI IS: 1/18 ECP-treated patients • Delayed CNI IS ≤8 days: 11/18 (61.2%) • Delayed CNI IS >8 days: 6/18 (33.3%)

CNI, calcineurin inhibitors; ECP, extracorporeal photopheresis; IS, immunosuppression; MMF, mycophenolate mofetil; NR, not reported.

^a^Only treatments where a change in dosage is reported here with any treatment not listed remained unchanged.

Leroux et al. (2022) compared forced expiratory volume (FEV_1_) evolution, indicative of BOS diagnosis and staging, in ECP-treated versus non-ECP treated patients among BOS recipients in a retrospective single-center study (44). Within 24 months following ECP initiation, five patients (62.5%) displayed an increase in FEV_1_ compared with the value at ECP onset, with two patients (25%) remaining stable and one (12.5%) displaying a decrease in FEV_1_. The study concluded that ECP aided the stabilization of lung function decline and therefore may be a useful therapeutic option for patients with BOS.

Idiopathic hyperammonemia and hypercapnic respiratory failure are toxic side effects of the CNIs cyclosporin and tacrolimus that affect lung transplant patients. A recent case report by Steinack et al. (2020) considered the potential of ECP as a second-line immunomodulatory therapy in early post-transplant cases where standard IS causes severe collateral damage (43). In this case report, ECP was used early after lung transplantation to treat idiopathic hyperammonemia and hypercapnic respiratory failure, likely caused by the toxicity of standard IS. Intensive early post-operative standard IS for the patient was reduced within one week of ECP treatment initiation to maintenance level IS; methylprednisolone was reduced from 30 to 25 mg. Treatment with MMF did not change. Follow-up for this patient lasted six months after the cessation of one year of ECP treatment, in which time the patient’s lung allograft function remained stable.

### Kidney transplantation

Case studies currently comprise the majority of available published evidence on the use of ECP in kidney transplantation, and only a small subset of these report IS-modifying outcomes related to the use of ECP (**Table 1**) (27). There is, however, a particularly high unmet need in kidney transplantation with no standard of care for chronic active rejection (62, 63). There are also no guidelines currently available for use of ECP in kidney transplantation.

One of the earliest case reports of ECP for treatment of recurrent rejection was published by Dall’Amico et al. (1998) and reports IS-modifying outcomes in renal transplant recipients (45). Of the four patients included in this study, ECP permitted a reduction of steroid use in three (mean ± SD; dosage pre-ECP: 16 ± 11 mg; dosage 6 months post-ECP: 8.5 ± 1 mg). Cyclosporine A and azathioprine doses were maintained during ECP treatment. Additionally, ECP was associated with improvement in glomerular filtration rate (GFR) in three ECP-treated patients and stabilization in the fourth patient.

Jardine et al. (2009) report a ten patient prospective case series where ECP was used to treat recurrent rejection and was associated with resolved rejection in all cases (12). Additionally, ECP enabled a reduction in overall immunosuppressive load with the dose for standard IS reduced following ECP initiation (**Table 2**). This reduction included both a reduced steroid pulse dose (mean; pre-ECP dose: 4.78 g/patient; post-ECP dose: 0.38 g/patient; n=10) and fewer days on antilymphocyte therapy (mean; pre-ECP: 15.4 days; post-ECP: 0.0 days; n=10).

Kumlien et al. (2005) reported the outcomes of seven patients with acute refractory renal graft rejection treated with ECP following lack of response to conventional antirejection treatment (46). At final follow-up (9–43 months) all patients had functioning grafts and five saw improvement in renal function, while in the remaining two patients renal function stabilized.

A multi-center retrospective study by Tamain et al. (2019) included 33 kidney transplantation recipients, one of the largest sample sizes investigating ECP in adult kidney transplant recipients (47). While modifications to standard immunosuppressive therapies were not specific outcomes included in this study, the indications for ECP use in kidney allograft rejections included patients that were resistant to standard therapies (n=18) or those for whom standard therapies were contraindicated due to active infections or cancers (n=15). Five patients in the study were treated by ECP as a single therapy, with all five still having a functional graft at 12 months. Outcomes beyond 12 months were more variable, with functional grafts at 24 months (n=2) and 72 months (n=1); one patient died with a functional graft at 22 months and the final patient did not recover their kidney function. The other 28 patients received ECP therapy along with standard IS treatments. Of all ECP-treated patients, 20 (61%) had a functional graft and 11 (33%) had a stabilization of kidney function at 12 months following ECP initiation. The study noted some efficacy limitations in certain patient groups, such as those with poor renal function at treatment initiation or those with long delays between rejection and treatment initiation; however, this is consistent with limitations of other standard IS therapies.

Augusto et al. (2021) presented three case studies on the use of ECP to treat AR in kidney transplant patients who had reduced standard IS following PTLD diagnosis, measuring kidney function, donor-specific antibody (DSA), and graft histology (48). The first patient started ECP three months after developing PTLD, yet had to stop after 16 sessions because of vascular access failure; however, complete AR resolution was still achieved. The other two patients were both initially treated with rituximab, amongst other IS therapies, following development of Epstein-Barr virus (EBV)-induced PTLD. Extracorporeal photopheresis was then introduced to the treatment regimen after persistence of AR. Both patients experienced favorable long-term follow-up with stable kidney function, undetectable DSA and no PTLD recurrence. ECP was found to be beneficial for controlling AR in all cases with favorable outcomes in all three patients. Initial observations indicated that ECP treatment should be initiated during the early stages of rejection.

In a case series of four kidney transplant patients, all of whom were considered as high immunological risk recipients due to previous transplantation or calculated PRA I +II levels of >50%, three patients presented stabilization of renal function during ECP treatment (64). In the fourth patient, no improvement was observed following ECP treatment and the patient experienced a progression to kidney graft failure. In two cases where patients had concomitant infections preventing administration of standard IS therapy, ECP was introduced alongside other therapeutic measures, enabling reduction of IS as well as stabilization of renal function. However, in one of these patients, treatment had to be subsequently suspended for logistical reasons and graft function worsened again.

### Liver transplantation

Liver transplantation is a well-established intervention for patients with irreversible liver failure or liver cancer, however the potential benefit of transplantation may vary depending on the cause (65). Although survival following liver transplantation has improved over time, late graft loss due to disease recurrence or chronic rejection can occur, with rates of loss varying due to etiology and post-transplant recidivism due to alcohol abuse (65).

While standard IS appears to be a successful management strategy in liver transplant recipients, a possible IS-modifying role of ECP has been investigated. A prospective single-center study was conducted in Italy in patients perceived to be at high risk of renal and neurological complications (49). The study investigated the use of ECP to avoid CNIs during the early post-operative treatment course. A full CNI-modifying regimen was possible in 5.5% of ECP-treated patients, while 33.3% were able to delay CNI by >8 days and 61.2% by ≤8 days as compared with controls. ECP was well tolerated by all treated patients, with no major side effects observed. Additionally, ECP did not appear to increase the risk of infectious complications, therefore sparing patients from antiviral and/or antibacterial prophylaxis. Overall, there was a statistically significant higher rate of survival in the ECP-treated group; 1-, 6-, and 12-month Kaplan-Meier estimates of patient survival were 94.4%, 88.1%, and 88.1% in the ECP group as compared with 94.4%, 77.7%, and 72.2% in the control group (*P*<0.0001).

A series of studies have investigated the use of ECP following liver transplantation using three protocols; the ‘avoiding CNI protocol’, the ‘ABO-incompatible protocol’, and the ‘hepatitis C virus (HCV)-positive protocol’ (66). Investigation into the HCV protocol was subsequently paused due to the introduction of direct antiviral drugs, however, preliminary data from the other two protocols support the theory that ECP can provide immunomodulation with low complication rates in terms of both survival and quality of life (67). The ‘avoiding CNI protocol’, introduces ECP as an alternative form of IS in the immediate post-operative period in an attempt to reduce CNI-related mortality. While this study provided preliminary data on potential areas for the use of ECP for immunomodulation, these results require further consideration. The ABO-incompatible protocol’ was developed for use in patients who rapidly deteriorate due to hepatic failure. At the final follow up of the preliminary trial (mean follow-up: 2836 days [range: 706–4335]), none of the 12 patients receiving ECP had developed an acute rejection (67).

## Discussion

Despite advances in IS therapy regimens over recent years, there are still key challenges for IS in the field of SOT, in both the overall effectiveness of standard IS therapies and their associated side effects (68). Taken together, the evidence discussed in this review suggests that ECP can be used to address these challenges in the context of both rejection prophylaxis and rejection treatment. The evidence level for the use of ECP varies widely, with the most evidence available in cardiothoracic transplantation, and the least in kidney transplantation. However, in all indications there is a clear need for high quality evidence from large RCTs. Greater understanding of ECP’s mechanism of action in SOT is also required to further support the development of ECP protocols and guidelines.

Although ECP has demonstrated potential across all the organ types discussed in this review, there are differences in the specific risks and complications associated with each organ type. Consequently, there is a clear need for organ-specific data and protocols.

There are variable guidelines and protocols on the use of ECP across countries, and some indications do not have established guidelines, likely due to the lack of RCTs. ECP regimens may also differ between difference centers in terms of treatment protocols which may impact patient outcomes between centers. However, suggested treatment protocols have been published which support attempts to harmonize the regimens used between centers (69, 70). ECP may also have potential uses in SOT beyond the current recommendations. For example, ECP may lead to reduced incidence of, or increased protection against, post-transplant viral infections and their associated complications, such as BK virus nephropathy or EBV-associated PTLD. Further research across all organ types is required to understand the full extent of ECP’s potential for clinical efficacy and confirm an acceptable safety profile.

This review highlights the IS-modifying potential of ECP in the four most common SOT procedures: heart, lung, kidney, and liver transplantation. Current IS strategies, such as CNIs, incur significant side effects that lack effective minimization therapies. ECP could play a substantial role in lowering the dosage of standard IS interventions, for example in CNI therapy, and therefore in limiting these side effects (49). Based on the scarce published literature, ECP appears to be predominantly used in cardiothoracic rather than abdominal SOT. However, research in all organ types suffers from insufficient RCT evidence, relying primarily on anecdotal data; further high-quality research is therefore required to elucidate the potential of ECP in the area of SOT.

### Strengths and limitations

This review presents a comprehensive overview of studies on IS-modifying properties of ECP across organ types across several decades of research.

A key limitation of this field is the lack of evidence provided by RCTs; it is challenging to draw generalized conclusions from the limited data available. The studies discussed in this review include case reports, case series and non-randomized cohort studies, and represent a maximum evidence level of three according to the Oxford Centre for Evidence-Based Medicine criteria (50). Finally, while available data support ECP for IS modification in kidney transplantation (47), there are limited published data on its use, safety, and efficacy in this indication. There is a clear need for further research in this area to support evidence-based decision-making, particularly in the context of kidney and liver transplantation.

## Conclusion

Due to a lack of effective strategies to reduce the side effects associated with the current standard of care IS, there is a key unmet need for patients following SOT. Current evidence supports the use of ECP for IS modification following SOT in multiple organ types, however there is a need for further high-quality research, in particular RCTs, in this area.

## Author contributions

CB: Conceptualization, Writing – review & editing. JA: Conceptualization, Writing – review & editing. FD: Conceptualization, Writing – review & editing. AZ: Conceptualization, Writing – review & editing.
